# Improving the Performance of BaMnO_3_ Perovskite as Soot Oxidation Catalyst Using Carbon Black during Sol-Gel Synthesis

**DOI:** 10.3390/nano12020219

**Published:** 2022-01-10

**Authors:** Verónica Torregrosa-Rivero, María-Salvadora Sánchez-Adsuar, María-José Illán-Gómez

**Affiliations:** Carbon Materials and Environment Research Group, Department of Inorganic Chemistry, Faculty of Science, Universidad de Alicante San Vicente del Raspeig, 03690 Alicante, Spain; vtr7@gcloud.ua.es (V.T.-R.); dori@ua.es (M.-S.S.-A.)

**Keywords:** BaMnO_3_ perovskite, diesel soot oxidation, sol-gel synthesis, carbon black

## Abstract

A series of BaMnO_3_ solids (BM-CX) were prepared by a modified sol-gel method in which a carbon black (VULCAN XC-72R), and different calcination temperatures (600–850 °C) were used. The fresh and used catalysts were characterized by ICP-OES, XRD, XPS, FESEM, TEM, O_2-_TPD and H_2-_ TPR-. The characterization results indicate that the use of low calcination temperatures in the presence of carbon black allows decreasing the sintering effects and achieving some improvements regarding BM reference catalyst: (i) smaller average crystal and particles size, (ii) a slight increase in the BET surface area, (iii) a decrease in the macropores diameter range and, (iv) a lower temperature for the reduction of manganese. The hydrogen consumption confirms Mn(III) and Mn(IV) are presented in the samples, Mn(III) being the main oxidation state. The BM-CX catalysts series shows an improved catalytic performance regarding BM reference catalyst for oxidation processes (NO to NO_2_ and NO_2_-assisted soot oxidation), promoting higher stability and higher CO_2_ selectivity. BM-C700 shows the best catalytic performance, i.e., the highest thermal stability and a high initial soot oxidation rate, which decreases the accumulation of soot during the soot oxidation and, consequently, minimizes the catalyst deactivation.

## 1. Introduction

Pollution generated by mobile sources is one of the main problems in urban areas as a consequence of the huge increase in the amount of on-road automobiles. The automotive exhaust is typically composed of nitrogen oxides (NO_x_), hydrocarbons (HC), carbon monoxide (CO) and soot (PM) that cause some well-known negative effects on the environment and human health. To regulate the automotive exhaust composition, in Europe, the EuroVI protocol establishes that, since 2015, the levels of NO_x_ and PM have to be reduced by 87% and 96%, respectively. To meet this regulation, the reduction of NO_x_ is performed by Selective Catalytic Reduction (SCR) and Lean NO_x_ Traps (LNTs), catalytic after-treatment systems [1,2]. For soot, all the new vehicles must be equipped with a unit based on a Diesel Particulate Filter (DPF), as the Catalyzed Continuous Regenerating Trap proposed by Johnson Matthey. Recently, to decrease the volume and the cost of after-treatment systems, combined technologies were also proposed, for example, using DPF-LNT/SCR systems in a different configuration in order to advantageously combine concentration and/or temperature gradients generated by the catalytic system. Thus, many types of catalysts were investigated for the simultaneous control of NO_x_ and soot, not only PGM-based systems (platinum-group metals such as Pt, Pd, Rh and Ir) but also other materials based on metallic oxides (spinel-type oxides, hydrotalcites, rare earth metal oxides, etc.). Due to the high cost of PGM catalysts, the development of PGM-free catalysts is one of the most challenging issues [3].

Perovskite-based catalysts (ABO_3_) are being deeply studied as promising alternatives to PGM-catalysts for automotive exhaust control because their properties are tunable by selecting: (i) the synthesis route, which will determine the morphology, textural properties, and crystallinity, and (ii) the composition, by modifying A/B cations or partially substituting A/B cations. The high number of possibilities allows the modification of the redox properties and, consequently, the catalytic performance [4,5,6,7,8].

However, as it is well known [4,5,6,7,8], the principal disadvantage of perovskite-like materials is their low BET surface area because, as ceramic oxides, perovskites are non-porous materials [7,8]. This fact highly impacts the intrinsic activity of perovskites and, by increasing the surface area, the performance of some perovskite-like catalysts would be comparable to PGM catalysts. Consequently, a challenging issue in the design of perovskite-like materials with high surface area [9] by different synthesis methods [10,11,12,13,14,15,16,17,18] such as the modified sol-gel synthesis [10], solvothermal methods [11], the synthesis of nanoparticles [9,10,12], and of nanoporous [10,13], mesoporous [14,15] and macroporous [16,17] perovskites. Among others, the nano casting technique allows the increase in the surface area using ordered mesoporous materials, as silica or carbon [9,18]. Interconnected structures facilitate the formation of a stable replica, and the pore structure and the particle morphology will depend on the selected material [10,14,15,19,20,21,22].

In a previous study [23], a BaMn_1−x_CuxO_3_ catalysts series was optimized as catalysts for NO_x_-assisted diesel soot oxidation by partial substitution of Mn for Cu, being BaMnO_3_ and BaMn_0.7_Cu_0.3_O_3_ the most active catalysts, even though only the latter was stable during successive soot oxidation cycles in TPR conditions. Thus, the aim of this paper is the development of BaMnO_3_ soot-oxidation catalysts with improved physical and chemical properties obtained by a modified sol-gel method using a hard template. The selection of the hard template was based on a preliminary study (summarized in Appendix A) from which it is concluded that the use of silica (SBA-15) as the hard template is forbidden as the strong interaction between the silica and barium (forming several barium silicates) avoids the formation of BaMnO_3_ perovskite as the main crystal phase. Thus, considering the positive effect on catalytic performance for soot oxidation of using model soot (Printex-U) for the synthesis of CeO_2_ support by solution combustion method [24], a carbon black (VULCAN XC-72R) was selected as a hard template to improve the performance of BaMnO_3_ solids as catalysts for NO to NO_2_ and NO_x_-assisted diesel soot oxidation. Additionally, based on the work of S. Zhuang et al. [10], the effect of the calcination temperature, ranging from the temperature required to decompose the hard template (600 °C) to the needed for the formation of the BaMnO_3_ hexagonal perovskite by the conventional sol-gel method (850 °C), was analyzed.

## 2. Materials and Methods

### 2.1. Synthesis and Characterization of Catalysts

A series of BaMnO_3_ solids were prepared by a modified sol-gel method in which carbon black (VULCAN XC-72R, as hard template), and different calcination temperatures (BM-CX, where X indicates the calcination temperature) were used. As a reference, a BaMnO_3_ solid was prepared by the conventional sol-gel synthesis (BM).

The sol-gel synthesis of BM [20] starts with a citric acid solution (1M) heated up to 60 °C and a pH of 8.5, adjusted with an ammonia solution, in which the precursors (Ba(CH_3_COO)_2_ and, Mn(NO_3_)_2_·4H_2_O) are added. The solution is heated up to 65 °C and after 5 h a gel is formed, which is dried at 90 °C for 48 h. Finally, the dried solid is calcined at 150 °C (1 °C/min) for 1 h, and at 850 °C (5 °C/min) for 6 h.

The modified sol-gel synthesis follows the steps of the conventional sol-gel synthesis above described but adds carbon black (Vulcan XC-72R) with a mass ratio 1:1 (carbon black: BaMnO_3_). Then, the mixture is vigorously agitated for 1 h, and after drying the mixture at 90 °C for 48 h, the solid is calcined at 150 °C (1 °C/min) for 1 h, and at different temperatures ranging from 600 °C to 900 °C (5 °C/min) for 6 h.

For sample characterization, different techniques were used.

The barium, manganese and copper content were measured by micro-X-ray fluorescence (μ-XRF), using an Orbis Micro-XRF Analyzer from EDAX and by ICP-OES, on a Perkin–Elmer device model Optimal 4300 DV. For ICP-OES analysis, the elements are extracted by the mineralization of the samples using a diluted aqua regia solution (HNO_3_:HCl, 1:3) and stirring at room temperature for 1 h.

The textural properties were determined by N_2_ adsorption at −196 °C using an Autosorb-6B instrument and by Hg porosimetry carried out in a Poremaster-60 GT equipment, both from Quantachrome (Anton Paar Austria GmbH). The samples were degassed at 250 °C for 4 h before the N_2_ adsorption experiments and dried at 60 °C for 12 h before Hg porosimetry analysis.

The degree of removal of carbon black used in the modified sol-gel synthesis was determined by Thermogravimetric Analysis (TGA) in a Q-600-TA equipment from Balzers Instruments (Pfeiffer Vacuum GmbH, Germany), by heating 10 mg of solid from room temperature to 950 °C (10 °C/min), under a flow of 100 mL/min of helium.

The crystalline structure was studied by X-ray Diffraction (XRD). The X-ray patterns were recorded between 20–80° 2θ angles with a step rate of 0.4°/min and using Cu Kα (0.15418 nm) radiation in a Bruker D8-Advance device.

The morphology of catalysts was analyzed by electronic microscopy, using a JEOL JEM-1400 Plus TEM equipment for Transmission Electronic Microscopy (TEM), and a ZEISS Merlin VP Compact for Field Emission Scanning Electronic Microscopy (FE-SEM).

The surface chemistry was assessed by X-ray Photoelectron Spectroscopy (XPS) using a K-Alpha Photoelectron Spectrometer by Thermo-Scientific with an Al K_α_ (1486.7 eV) radiation source. To obtain XPS spectra, the pressure of the analysis chamber was maintained at 5 × 10^−10^ mbar. The binding energy (BE) and kinetic energy (KE) scales were adjusted by setting the C1s transition at 284.6 eV, and the BE and KE values were then determined with the peak-fit software of the spectrometer. The XPS ratios O_Lattice_/(Ba + Mn) and Mn(IV)/Mn(III) were calculated by the area under the suggested deconvolutions of O1s, Mn 3p^3/2^ and Ba 3d^5/2^ bands.

Reducibility of catalysts was determined by Temperature Programmed Reduction with H_2_ (H_2_-TPR) in a Pulse Chemisorb 2705 (from Micromeritics) with a Thermal Conductivity Detector (TCD) and using 30 mg of sample which was heated at 10 °C/min from 25 °C to 1000 °C in 5% H_2_/Ar atmosphere (40 mL/min). The quantification of the H_2_ consumption was carried out using a CuO reference sample.

O_2_-TPD experiments were performed in a TG-MS (Q-600-TA and Thermostar from Balzers Instruments (Pfeiffer Vacuum GmbH, Germany) respectively), with 16 mg of sample heated at 5 °C/min from room temperature to 900 °C under a 100 mL/min of helium atmosphere. The 18, 28, 32 and 44 *m*/*z* signals were followed for H_2_O, CO, O_2_ and CO_2_ (respectively) evolved during these experiments. The amount of evolved oxygen is estimated using a CuO reference sample.

### 2.2. Activity Tests

The activity for NO and NO_x_-assisted soot oxidation was carried out by Temperature Programmed Reaction in a quartz fixed-bed reactor, heated up from 25 °C to 800 °C (10 °C/min), under a gas flow mixture (500 mL/min) containing 500 ppm NO_x_, 5% O_2_, balanced with N_2_. For NO oxidation experiments, 80 mg of catalyst was diluted with 320 mg SiC. Soot oxidation tests were performed mixing 80 mg of catalyst and 20 mg of Printex-U (the carbon black used as model soot) with a spatula to ensure loose contact, and the mixture was diluted with 300 mg of SiC. The most active catalysts were also tested in isothermal soot oxidation conditions, at 450 °C for 180 min. The gas composition was monitored by specific NDIR-UV gas analyzers for NO, NO_2_, CO, CO_2_ and O_2_ (Rosemount Analytical Model BINOS 1001, 1004 and 100, Emerson Electric Co., St. Louis, MO, USA).

The NO_x_ conversion and the NO_2_ generation percentages were calculated using the following equations:

(1) $${\mathrm{NO}}_{x}\;\mathrm{conversion}\left(\%\right)=\frac{\left({\mathrm{NO}}_{x,\mathrm{in}}-{\mathrm{NO}}_{x,\mathrm{out}}\right)}{{\mathrm{NO}}_{x,\mathrm{in}}}\cdot 100$$

(2) $${\mathrm{NO}}_{2,\mathrm{out}}/{\mathrm{NO}}_{x,\mathrm{out}}\left(\%\right)=\frac{{\mathrm{NO}}_{2,\mathrm{out}}}{{\mathrm{NO}}_{x,\mathrm{out}}}\cdot 100$$

where NO_2,out_ and NO_x,out_ are the NO_2_ and NO_x_ (NO + NO_2_) concentrations measured at the reactor exit.

The soot conversion and CO_2_ selectivity were determined as:

(3) $$\mathrm{Soot}\;\mathrm{conversion}\;\left(\%\right)=\frac{\sum_{0}^{t}{\mathrm{CO}}_{2}+\mathrm{CO}}{\sum_{0}^{\mathrm{final}}({\mathrm{CO}}_{2}+\mathrm{CO})}\cdot 100$$

(4) $${\mathrm{CO}}_{2}\;\mathrm{Selectivity}\;\left(\%\right)=\frac{{\mathrm{CO}}_{2}}{\sum_{0}^{\mathrm{final}}({\mathrm{CO}}_{2}+\mathrm{CO})}\cdot 100$$

where $\overset{t}{\underset{0}{\sum }}({\mathrm{CO}}_{2}+\;\mathrm{CO})$ is the amount of CO_2_ and CO evolved at a time t, while $\overset{\mathrm{final}}{\underset{0}{\sum }}{\mathrm{CO}}_{2}$ and $\overset{\mathrm{final}}{\underset{0}{\sum }}({\mathrm{CO}}_{2}+\;\mathrm{CO})$ are the total amount of CO_2_ and CO + CO_2_ evolved during the test.

## 3. Results and Discussion

### 3.1. Characterization of Fresh Catalysts

**Figure A5:**
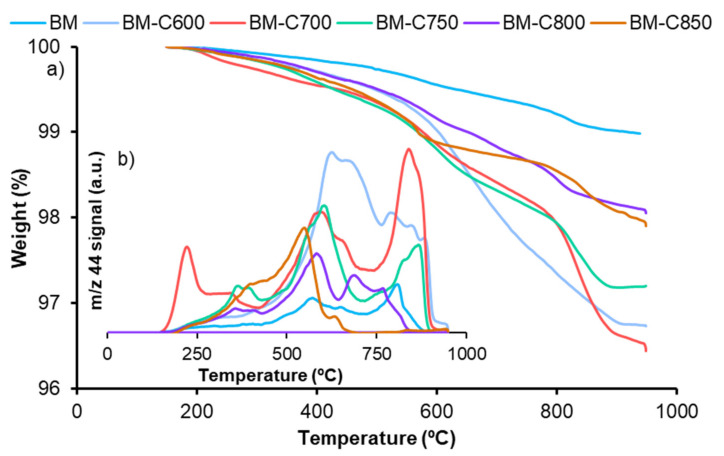
(**a**)TGA profiles and (**b**) 44 m/z signal during the analysis of BM-CX series.

The remaining carbon black after the calcination step was determined by thermogravimetric analysis. The weight profiles for the BM-CX series (shown in Figure A5 in Appendix B), indicate that most of the carbon black was efficiently removed during the synthesis as the percentage of remaining carbon black ranges from 3% for BM-C600 to 1% for BM-C850 (see data in Table A3 in Appendix B) [10].

#### 3.1.1. Structural Properties: XRD

**Figure 1:**
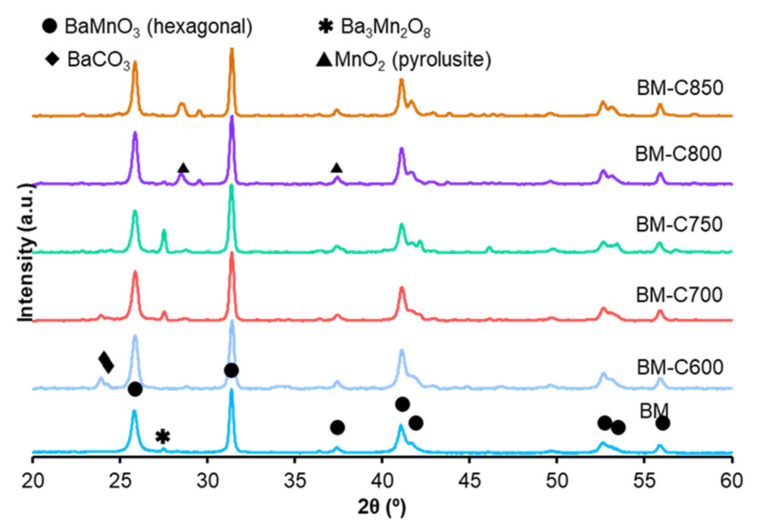
X-ray patterns of BM-CX series and BM as reference.

X-ray patterns for the BM-CX series and BM reference are shown in Figure 1. As it is expected [10], the use of the modified sol-gel synthesis allows a decrease in the calcination temperature required to achieve the perovskite-like structure from 850 °C (employed in the conventional sol-gel synthesis) to 600 °C. The main crystalline phase for all the catalysts is the BaMnO_3_ hexagonal (PDF number: 026-0168, denoted by the ICDD, the International Centre of Diffraction Data) perovskite-like structure. This structure is formed by chains of face-sharing MnO_6_ units instead of being formed by corner-shared MnO_6_ units, as it is observed for most of the perovskite structures [25]. On the other hand, the minority phase composition is depending on the calcination temperature:

(i) at low calcination temperature (BM-C600 and BM-C700), some barium carbonate is present because the calcination step is performed in static air and the CO_2_ produced by the citric acid decomposition could remain in the samples calcined at low temperatures [26].
(ii) at intermediate calcination temperatures (BM-C700 and BM-C750), a low amount of barium-manganese oxide (Ba_3_Mn_2_O_8_) is identified.
(iii) at high calcination temperatures (BM-C800 and BM-C850), some manganese dioxide is formed.

The average crystal size for BM-CX series and BM reference catalyst, calculated by the Scherrer equation using the main peak of the BaMnO_3_ hexagonal phase (c.a. 31.4° corresponding to the (110) diffraction plane), is included in Table 1. The average crystal size for the BM-CX series decreases with the calcination temperature, due to the reduction of the sintering effects. Note that, although an increase with the calcination temperature is observed, the values are always lower than the corresponding to the BM reference catalyst. The lattice parameters indicate that the crystal structure is not significantly modified with respect to BM reference.

#### 3.1.2. Textural and Morphological Properties

**Figure 2:**
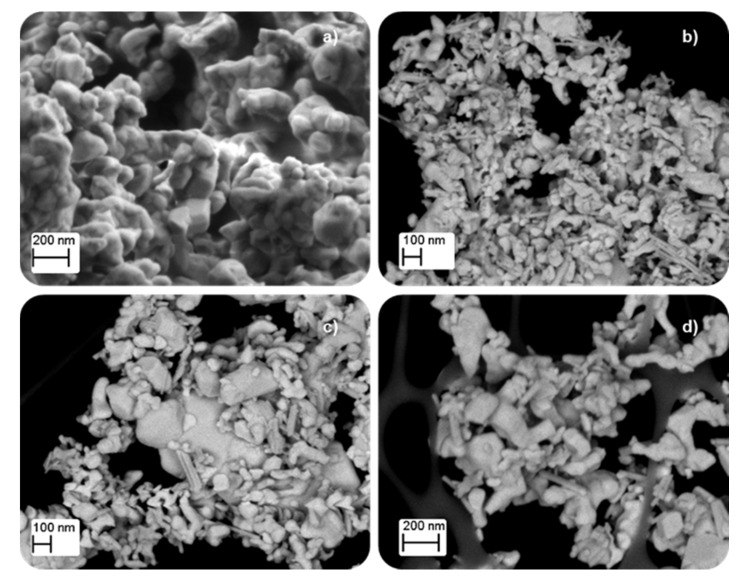
FE-SEM images of (**a**) BM, (**b**) BM-C600, (**c**) BM-C700, and (**d**) BM-C800.

**Figure 3:**
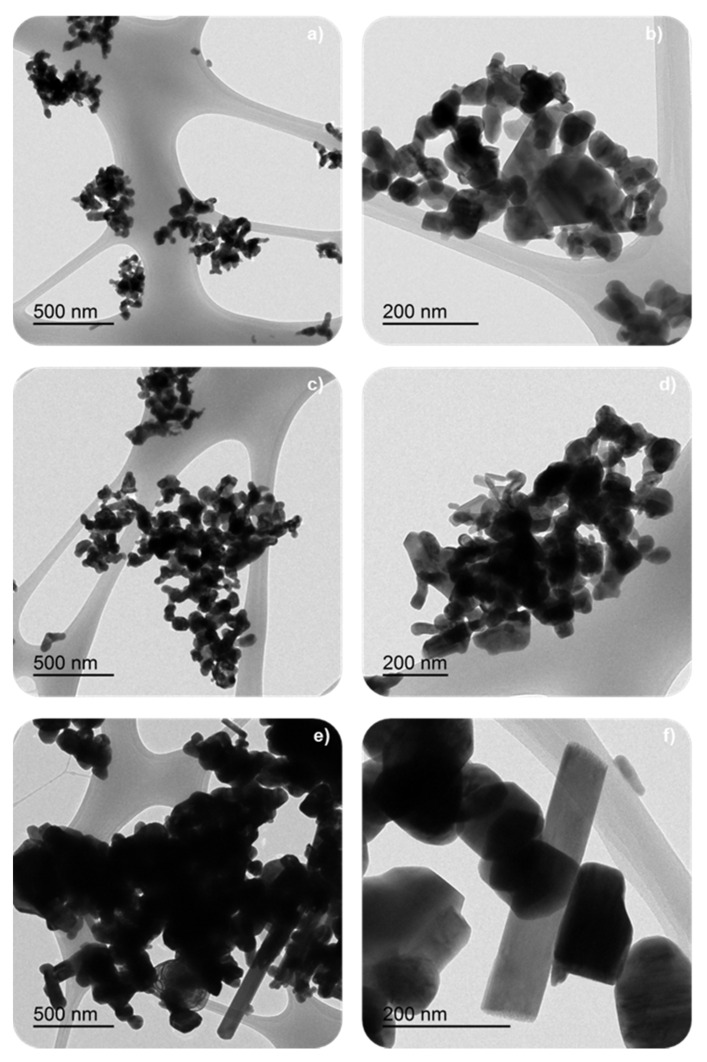
TEM images of some BM-CX catalyst series using different magnifications: (**a**,**b**) BM-C600, (**c**,**d**) BM-C700 and (**e**,**f**) BM-C800.

Table 1 shows the low BET surface area of samples, as it is expected for solids with poor porosity as mixed oxides with perovskite-like structures are [8,27,28,29]. However, the BET surface area shows a slight increase, and the corresponding average crystal size diminishes, as the calcination temperature decreases because of the minimization of sintering. It was reported that perovskite-like solids with a high surface area were obtained at calcination temperatures between 450 °C and 500 °C [9], so, as the minimum calcination temperature used was 600 °C, lower BET surface areas are expected for the BM-CX catalysts. The decrease in the average crystal size with the calcination temperature, and also in the average particle size, was observed in FE-SEM and TEM images, shown in Figure 2 and Figure 3, respectively. The images reveal the presence of amorphous particles, showing different sizes that difficult the accuracy in measuring average particle size which allows obtaining a particle size distribution.

**Figure 4:**
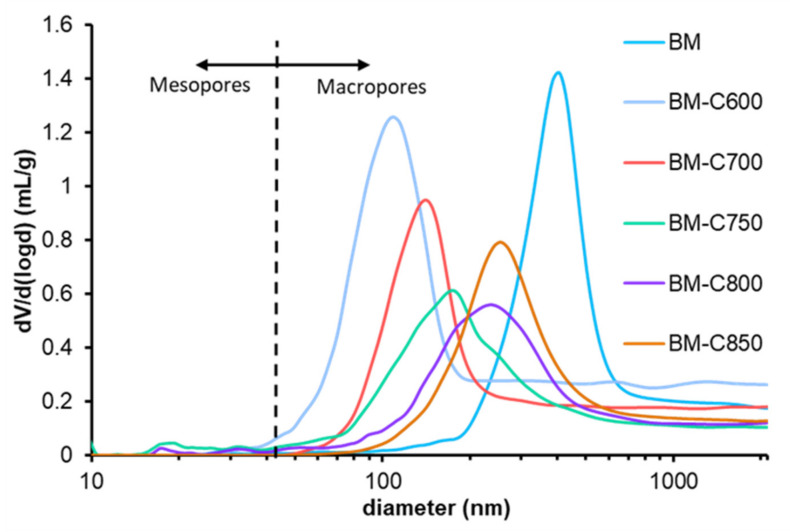
Logarithmic pore size distribution of the BM-CX series and BM.

The range of pore diameter was estimated by Hg porosimetry and Figure 4 features the logarithmic pore size distribution for the BM-CX series and BM reference catalyst. The data indicate that the catalysts are mainly macroporous and that the use of the carbon black allows developing a well-defined porosity if a low calcination temperature is used. Note that macropores of BM catalyst mainly correspond to the inter-particle space while for BM-CX catalysts series, the macropores are related to intra-particle space. Finally, it is confirmed that the sintering effects increase with calcination temperature [30].

In conclusion, the use of carbon black during sol-gel synthesis: (i) allows reducing the calcination temperature needed to achieve a perovskite-like structure and, (ii) the sintering effects are diminished at low calcination temperature, promoting lower average crystal size, smaller particles and an enhanced macroporosity [9,31,32].

#### 3.1.3. Surface Composition: XPS

**Figure 5:**
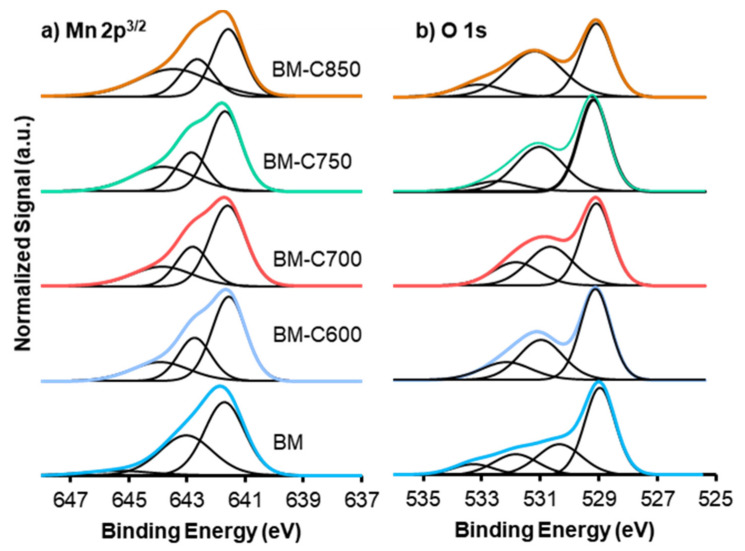
XPS spectra of (**a**) Mn2p3/2, and (**b**) O1s transitions of BM-CX series and BM as reference.

Figure 5 shows the XPS spectra recorded for (a) O1s and (b) Mn2p^2/3^ transitions. As XPS analysis provides information up to 3 nm in depth (with a spatial resolution of 200 μm [33]), the XPS data inform about the surface composition [34].

The deconvolution of the XPS signal for Mn reveals the presence of different oxidation states and/or different interactions with the bulk. The Mn2p^3/2^ spectra suggest the presence of Mn(III) and Mn(IV) [35]. The main signal, which presents an asymmetric shape, can be deconvoluted in three contributions: (i) the signal at higher binding energy (c.a. 645 eV) corresponds to the Mn(III) satellite peak since Mn(III) is paramagnetic [31], (ii) the peak at ca. 643 eV is assigned to Mn(IV), and (iii) the peak at c.a. 641 eV is associated to Mn(III) [31,32,33]. Table 2 includes the Mn(IV)/Mn(III) ratio, calculated using the area under the corresponding deconvoluted peaks. Most of the catalysts present an Mn (IV)/Mn(III) value lower than one, revealing that Mn(III) is the main oxidation state on the surface. However, the Mn(IV)/Mn(III) ratio points out an increment of Mn(IV) for the sample obtained at the highest calcination temperature (850 °C).

Three different contributions are identified for O1s XPS spectra, which are attributed to lattice oxygen (O_Lattice_, ca. 529 eV), oxygen from surface groups as hydroxyl and/or carbonates species (ca. 531 eV) [36,37] and oxygen from moisture (ca. 533 eV) [38,39]. The main contribution for BM is due to lattice oxygen, as it is observed in the literature for related manganites [19,38,39,40], while BM-CX catalysts show an increase in oxygenated surface groups. Therefore, the modified sol-gel synthesis seems to promote a different oxygen distribution on the surface. The O_Lattice_/(Ba + Mn) ratio reveals the presence of oxygen defects because the value is lower than the nominal one (1.5) [41] for all the BM-CX catalysts. Moreover, the ratio decreases as the calcination temperature increases. These vacancies/defects are generated to compensate for the positive charge defect due to the presence of Mn(III). Considering these results, an enhanced lattice oxygen mobility is expected for BM-CX mixed oxide regarding BM [42].

#### 3.1.4. Reducibility: H_2_-TPR

**Figure 6:**
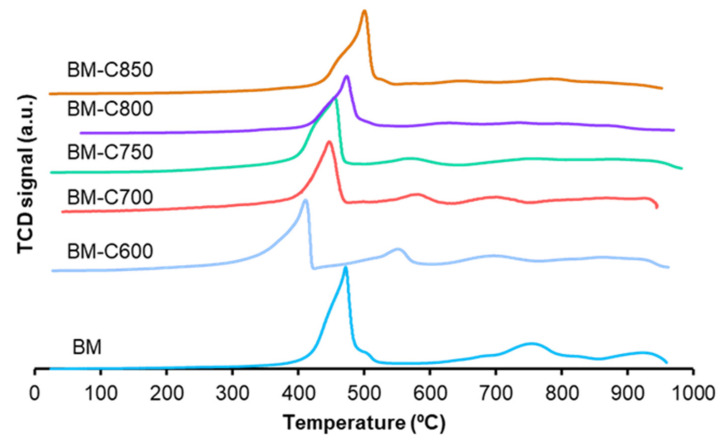
Hydrogen consumption profiles in TPR conditions of BM-CX series and references.

To study the reducibility of the catalysts, Temperature Programmed Reduction in hydrogen experiments was carried out. Figure 6 shows the hydrogen consumption profiles, which present three regions:

(i) At low temperature, among 300–600 °C, Mn(IV) is reduced to Mn(II) in two steps; firstly Mn(IV) is reduced to Mn(III) shown as a shoulder in the profiles, and, secondly, the most intense peak corresponds to Mn(III) reduction to Mn(II) [27,28,29,43] and its high intensity supports that the amount of Mn(III) is larger than Mn(IV), as the XPS data reveal for the surface (see Table 2).
(ii) At 550 °C, the H_2_ consumption could be related to the presence of the remaining carbon black because the intensity is higher for BM-C600, which is the sample presenting the highest amount of hard template after calcination (see Table A3 in Appendix B).
(iii) Between 600 °C and 800 °C oxygen species decompose.
(iv) At the highest temperatures, above 800 °C, bulk Mn(III) is reduced to Mn(II) [44] as several authors have concluded that the reduction up to Mn(0) is not achieved, being the final oxidation state Mn(II) [45,46,47]. This last contribution almost does not appear in BM-CX catalysts as the low average size of the particles (observed by microscopy) leads to a lower amount of bulk manganese.

A shift to lower temperatures of the reduction temperature for the maximum of the main reduction peak as the calcination temperature decreases is observed, which is due to the lower particle size that increases the number of surface species more likely to be reduced. Similar results were observed by S. Irusta et al. for LaMnO_3_ [48] and by Y. Gao et al. [49] for BaMnO_3_. Therefore, as the calcination temperature increases and the sintering effects become more evident, larger particles are formed (observed by TEM in Figure 4) and the temperature of the main reduction peak is shifted to higher temperatures.

**Figure 7:**
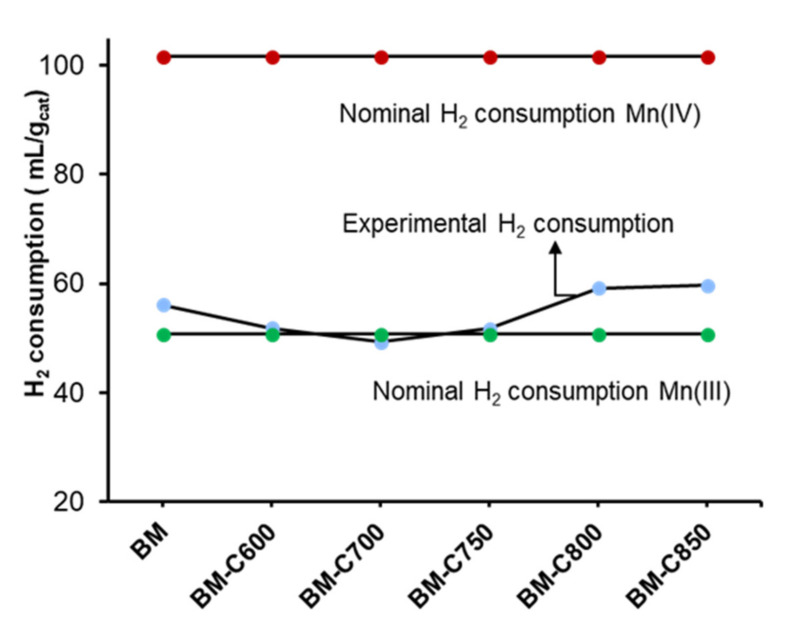
Hydrogen consumption between 150–500 °C, corresponding to manganese reduction.

The experimental hydrogen consumption per gram of catalyst was estimated for the region between 150 °C and 500 °C of the H_2_ consumption profiles shown in Figure 6. The nominal hydrogen consumption was calculated considering the total reduction of manganese only as Mn(IV) or Mn(III), and the obtained values are compared with the experimental ones in Figure 7. In this figure, if the experimental values are close to the maximum values (red points), it means that Mn(IV) is the main oxidation state, but if the values are close to the minimum ones (green points) it points out that Mn(III) is the predominant oxidation state or it could also mean that the reduction of manganese is not completed. Therefore, the results apparently show that Mn(III) is the main oxidation state in the perovskite, as XPS data suggest for the surface. However, this result could also mean that manganese is not being totally reduced during the H_2_-TPR experiment.

#### 3.1.5. Oxygen Desorption: O_2_-TPD

**Figure 8:**
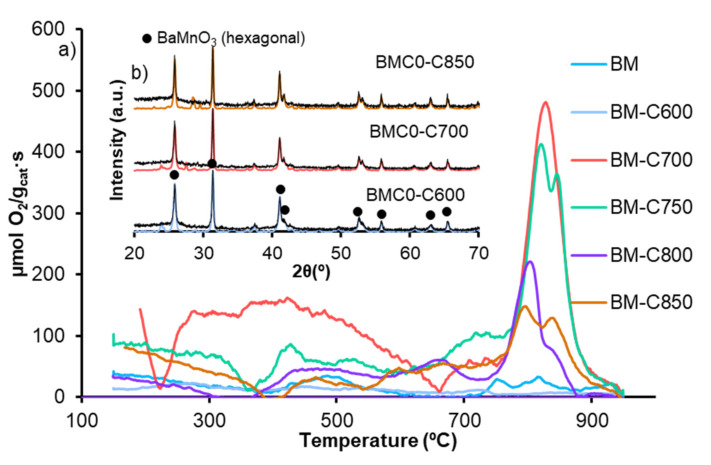
(**a**) O_2_-TPD profiles of BM-CX series and BM as reference and (**b**) X-ray patterns of fresh (colored line) and used (black line) BM-C600, BM-C700 and BM-C850 as representative BM-CX samples.

Figure 8 shows the profiles of oxygen evolved from BM-CX catalysts series and from BM reference catalyst during Temperature Programmed Desorption experiments. BM-CX catalysts mainly evolve oxygen at high temperatures (<700 °C), named β-O_2_, which comes from the perovskite lattice and is related with the Mn(IV) to Mn(III) reduction and the presence of oxygen defects that facilitate the desorption. Both characteristics boost lattice oxygen mobility [23,47]. The amount of β-O_2_ emitted, shown in Table 2, reveals that there is not a direct correlation with the calcination temperature as the amount of evolved β-O_2_ is higher than the corresponding to BM for all catalysts, except for BM-C600, but the values decrease for samples obtained at calcination temperatures higher than 700 °C. Note that the BM-C700 catalyst also evolves oxygen at low temperatures, named α-O_2_, which is enhanced by the presence of the oxygen adsorbed on surface defects/ vacancies [27,50], also detected by XPS (see Table 1). Thus, it seems that the ability to generate β-O_2_ (oxygen that comes from the perovskite lattice) relies on the number of oxygen vacancies/defects in the catalysts, but it also depends on the manganese reducibility. Therefore, BM-C700 shows the optimal combination between the number of oxygen vacancies and the reducibility (shown by H_2_-TPR) and consequently, it shows the highest oxygen mobility. It is remarkable that the perovskite-like structure is not destroyed after the O_2_-TPD analysis, since the corresponding X-ray patterns (Figure 8b) of used samples show the BaMnO_3_ hexagonal as the unique crystal phase, pointing out that Mn(III) and Mn(IV) are not reduced to metallic manganese.

In conclusion, the characterization results indicate that the use of low calcination temperatures in the presence of a carbon black (modified sol-gel synthesis) allows decreasing the sintering effects, therefore several improvements are achieved regarding BM reference catalyst: (i) smaller average crystal and particles size are obtained, (ii) the BET surface area slightly increases (from 5 m^2^/g to 20 m^2^/g), (iii) a decrease in the macropores diameter size range, (iv) the reduction of manganese occurs at lower temperatures, and (v) the hydrogen consumption confirms the presence of Mn(III) and Mn(IV) in the samples, also suggested by XPS for catalysts surface, being Mn(III) the main oxidation state. Finally, note that among the BM-CX catalysts series, BM-C700 combines high reducibility and a large number of surface oxygen defects that boost the oxygen mobility through the perovskite lattice.

### 3.2. Activity Tests

As BM-C700/BM-C750 and BM-C800/BM-C850 present similar physical and chemical properties, BM-C600, BM-C700 and BM-C850 were selected to determine the catalytic performance for NO and NO_x_-assisted soot oxidation.

#### 3.2.1. NO to NO_2_ Oxidation

**Figure 9:**
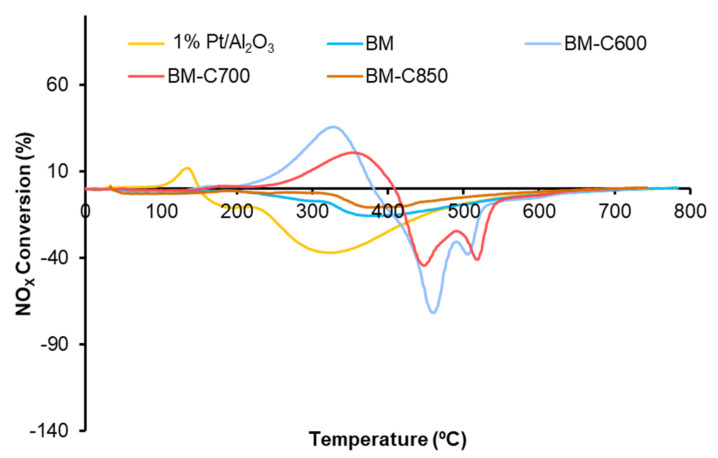
NO_x_ conversion profiles in NO_x_-TPR conditions of BM−CX series and references (1% Pt/Al_2_O_3_ and BM).

**Figure 10:**
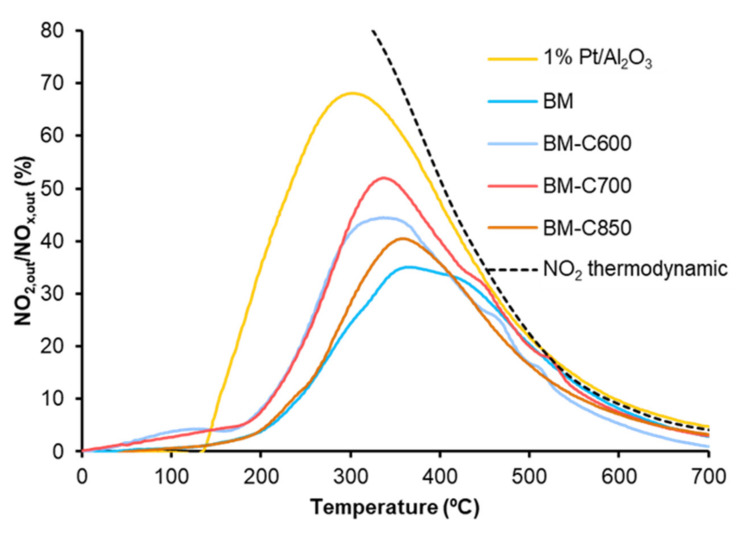
NO_2_ emission profiles in NO_x_-TPR conditions of BM-CX series, and references (1% Pt/Al_2_O_3_ and BM).

NO_X_-TPR tests are useful to determine the catalytic activity for the NO_x_ adsorption/desorption process and NO to NO_2_ oxidation. Figure 9 shows the NO_x_ conversion profiles in which, according to Equation (1), a positive signal is due to the adsorption of NO_x_ and a negative signal is related to the desorption of NO_x_. Although BM-C600 and BM-C700 catalysts seem to present some NO_x_ adsorption/desorption capacity, it is very low with respect to the observed for active catalysts for NO_x_-storage as BaTi_1-x_Cu_x_O_3_ solids [51]. The NO_x_ adsorption/desorption capacity is related to the presence of BaCO_3_, which is an active site for the NO_x_ adsorption process [52,53]. Therefore, the increase in the NO_x_ adsorption capacity as the calcination temperature decreases is directly related to the amount of BaCO_3_, identified by XRD (Figure 1). This fact was confirmed as, in a second consecutive NO_x_-TPR experiment (not shown), the catalysts do not show NO_x_ adsorption capacity because barium carbonate was decomposed during the first NO_x_-TPR. Due to the slight NO_x_ adsorption capacity observed for BM-C600 and BM-C700, the NO_2_ generation profiles, shown in Figure 10, do not show the total amount of NO_2_ generated, just the amount not adsorbed [51].

The NO_2_ emission profiles presented in Figure 10 reveal that all the catalysts are active for NO to NO_2_ oxidation below 500 °C (a temperature in the range of interest for practical application [54]) and that the catalysts obtained at low calcination temperature (BM-C600 and BM-C700) show a remarkable improvement regarding the BM reference catalyst. Note that BM-C700 shows the best catalytic performance as it features a high amount of oxygen vacancies and high reducibility that promote the highest lattice oxygen mobility.

Finally, although the addition of carbon black during the sol-gel synthesis allows the improvement in the catalytic activity, it is still lower than the shown by the platinum-based catalyst used as reference (1% Pt/Al_2_O_3_).

#### 3.2.2. NO_x_-Assisted Diesel Soot Oxidation

Due to the high catalytic activity for NO to NO_2_ oxidation, the samples were used as catalysts for NO_x_-assisted soot oxidation. The purpose is to check if the selected catalysts present an improved performance regarding the BM reference catalyst, which is active for this process but suffers a deactivation during successive NO_x_-TPR cycles [23].

**Figure 11:**
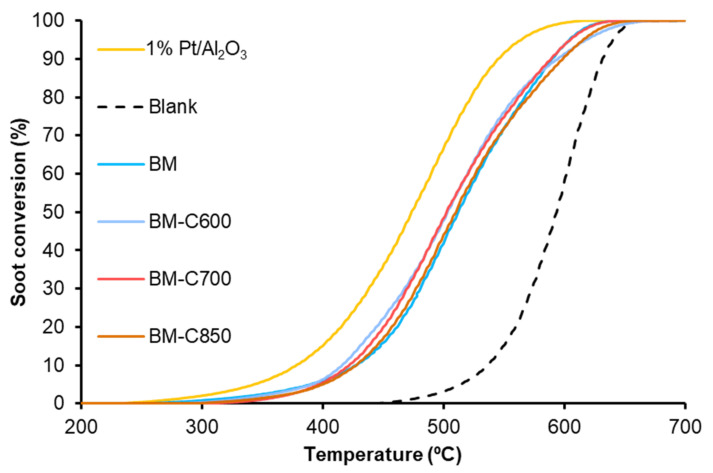
Soot oxidation profiles in NO_X_-TPR conditions of BM-CX series, and references (1% Pt/Al_2_O_3_ and BM).

**Figure 12:**
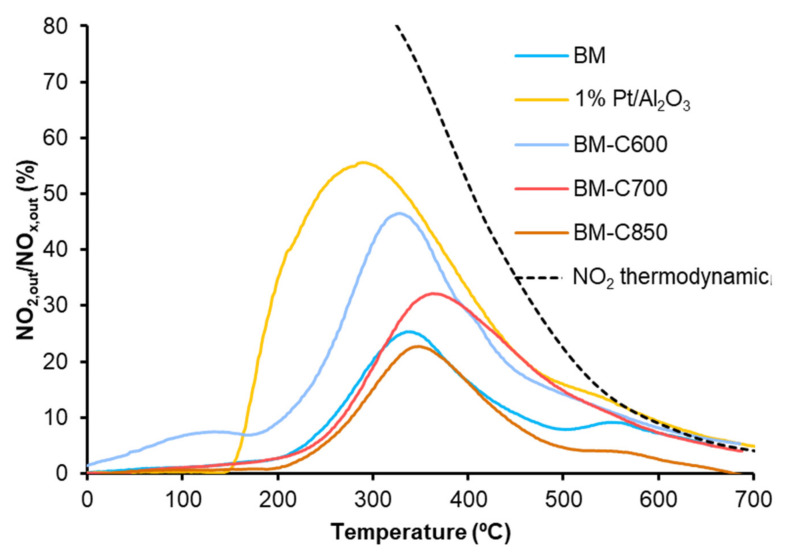
NO_2_ emission profiles during soot oxidation in NO_x_-TPR conditions of BM-CX series, and references (1% Pt/Al_2_O_3_ and BM).

The Temperature Programmed soot oxidation profiles, plotted in Figure 11, show that the BM-CX catalysts series is active since the soot oxidation is performed at a lower temperature than bare soot (blank). However, the presence of the carbon black and the calcination temperature do not affect the catalytic activity as all BM-CX catalysts present a similar performance to that of the BM reference. So, it seems that the improvement in the physical and chemical properties, mainly observed for BM-C700, does not improve the catalytic activity for soot oxidation in the NO_x_-TPR reaction conditions. Additionally, note that all the soot conversion profiles of BM-CX samples are close to the observed for the platinum-based catalyst used as reference. The obtained results are in good agreement with the NO_2_ generation profiles (Figure 10) because the maximum generation of NO_2_ determines the soot oxidation performance [55,56,57]. Thus, the most active catalyst is the platinum-based one, which shows the lowest temperature to achieve the maximum percentage of NO_2_. For BM-CX and BM catalysts the NO_2_ generation profiles are slightly shifted to higher temperatures, and the same trend is observed in the soot oxidation profiles. In fact, the comparison between NO_2_ emission profiles during NO_x_-TPR experiments (Figure 10) and the corresponding profiles during soot oxidation in NO_x_-TPR conditions (Figure 12) confirms that the soot oxidation process is carried out by NO_2_ reduction, being the difference between profiles the fraction of NO_2_ consumed. Finally, as it is expected for manganese-based perovskites [58,59,60], all the catalysts show a high CO_2_ selectivity (above 95%) during the NO_x_-TPR experiment. The electronic configuration of manganese as Mn(III) (d^4^) is optimal to interact with CO molecules and due to the lability of Mn(IV)-O bond, the CO oxidation is complete at rather low temperatures [49].

**Figure 13:**
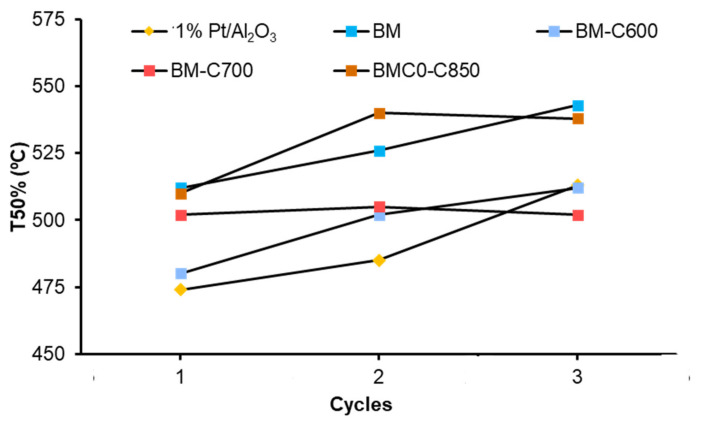
T50% values of BM-CX series, and references (1% Pt/Al_2_O_3_ and BM), during consecutive NO_x_-TPR cycles.

To study the stability of the catalysts, three consecutive NO_x_-TPR cycles of soot oxidation were carried out. Figure 13 summarizes the T50% values (which is the temperature required to achieve the 50% of soot conversion) in the successive cycles. All catalysts deactivate during successive NO_x_-TPR cycles, but BM-C700 shows the most stable performance. Therefore, although the BM-C700 catalyst shows a lower catalytic activity than the platinum-based catalyst used as a reference, the catalytic activity of the former remains after three consecutive cycles, while the platinum-based catalyst shows a deactivation, probably related to the sintering of platinum particles and/or to irreversible oxidation [61,62]. The same trend is observed for the CO_2_ selectivity, being higher than 95% during the successive NO_x_-TPR cycles for all tested BM-CX catalysts. Thus, it seems that the improved properties of BM-C700 boost a more stable performance during NO_x_-TPR cycles.

**Figure 14:**
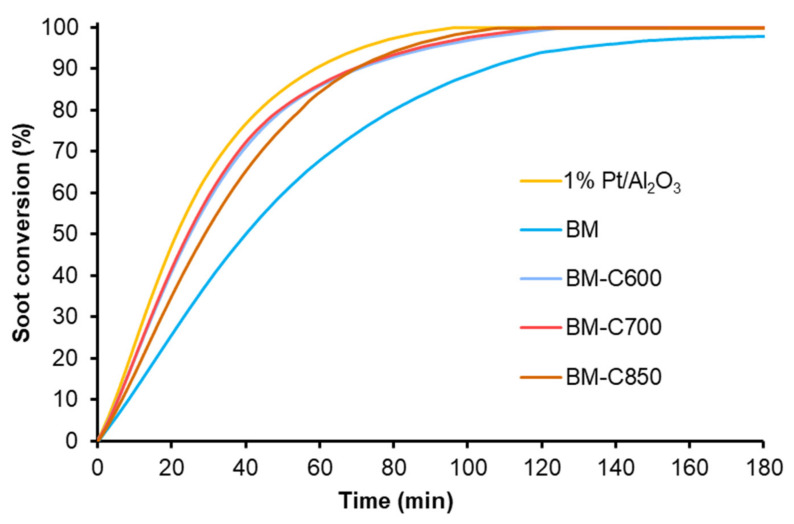
Soot conversion profiles at 450 °C of BM-CX series, and references (1% Pt/Al_2_O_3_ and BM).

In order to study the catalytic performance for soot combustion at temperatures in the range of interest of a diesel particulate filter, two consecutive isothermal experiments at 450 °C were carried out. Figure 14 shows the soot conversion profiles corresponding to the first isothermal reaction cycle for BM-CX catalysts, BM and the platinum-based catalyst used as references.

In general terms, the first isothermal soot oxidation cycle reveals that BM-CX catalysts show a higher catalytic activity at 450 °C than BM since BM is not able to totally remove the initial amount of soot after 180 min, while BM-CX samples require over 100 min to remove the 100% of the initial soot. The initial soot oxidation rates were calculated from the slope of soot conversion profiles during the first 20 min of the experiment and the values are included in Table 3, besides the CO_2_ selectivity. BM-CX catalysts present a higher initial soot oxidation rate than the BM reference, which is close to the observed for the platinum-based catalyst.

Finally, the soot oxidation profiles for the second successive isothermal test at 450 °C are similar to the shown in Figure 13 corresponding to the first reaction. The initial soot oxidation rate values included in Table 3 reveal that even working in cyclic conditions, the BM-CX catalysts are more active and stable than BM reference since the catalysts show an almost constant performance (initial soot oxidation rate and CO_2_ selectivity).

### 3.3. Characterization of Used Catalysts

The catalysts used in the activity tests (NO_x_-TPR and isothermal soot oxidation) were characterized by XRD, XPS and TEM, in order to check if the samples are modified during reactions.

**Figure 15:**
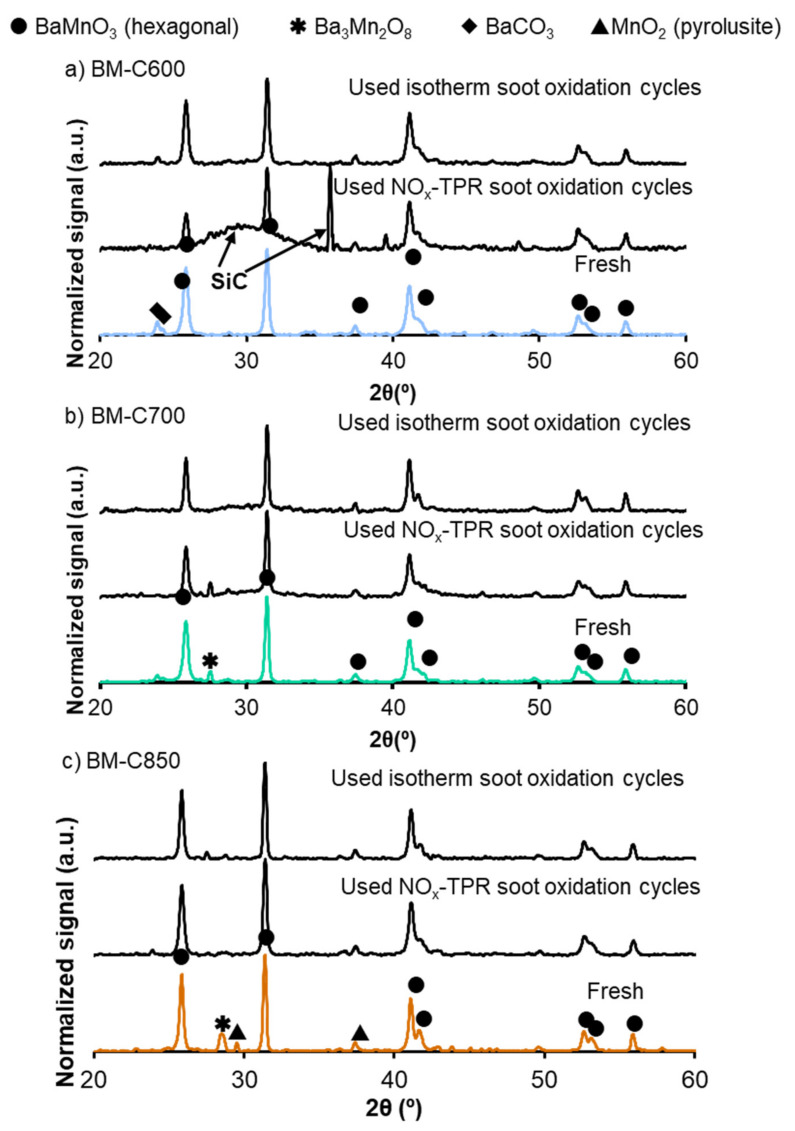
X-ray patterns of fresh and used BM-CX catalysts: after three NO_x_-TPR consecutive cycles and two isothermal soot oxidation cycles at 450 °C: (**a**) BM-C600, (**b**) BM-C700 and (**c**) BM-C850.

In Figure 15 the X-ray patterns of fresh and used catalysts are compared. The used samples hold the crystal structure after the oxidation processes, but, due to the high temperature reached during NO_x_-TPR soot oxidation (800 °C), the barium carbonate is present in the fresh BM-C600 and BM-C700 is removed. This fact explains the loss of the NO_x_ adsorption capacity during a second NO_x_-TPR cycle previously indicated. However, barium carbonate is not totally decomposed after isothermal tests due to the lower working temperature (450 °C). Moreover, during NO_x_-TPR and isothermal experiments the amount of minority crystal phases (Ba_2_Mn_3_O_7_ and MnO_2_) decreases, consequently, the used catalysts are more homogeneous than the fresh ones. Therefore, it is concluded from X-ray patterns that the selected BM-CX catalysts present a highly stable crystal perovskite-like structure.

The O_L_/(Mn + Ba) and Mn(IV)/Mn(III) ratios, calculated by XPS data for catalysts used in NO_x_-TPR and isothermal soot oxidation processes, are shown in Table 4. The comparison of these XPS data with the corresponding fresh samples reveals that the catalysts do not significantly modify the surface chemistry after being exposed to both experimental conditions (NO_x_-TPR and isothermal), being BM-C700 the catalyst with the most stable surface properties.

**Figure 16:**
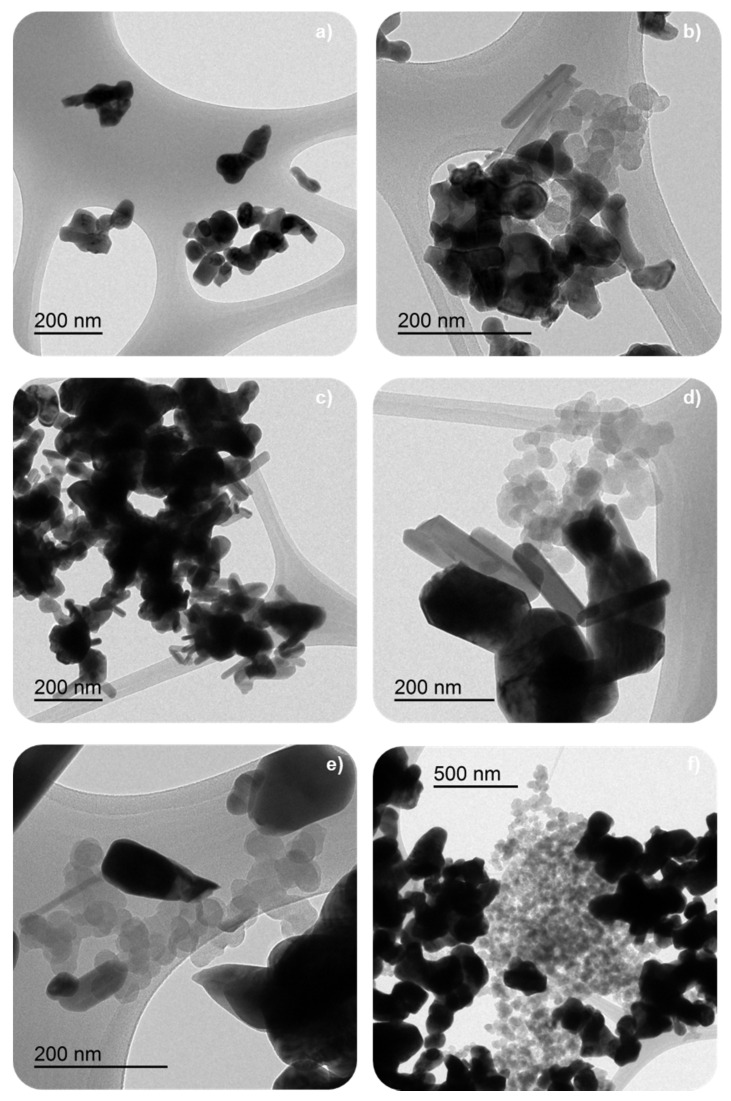
TEM images of selected BM-CX catalysts; from top to bottom (**a**,**b**) BM-C600, (**c**,**d**) BM-C700, and (**e**,**f**) BM-C850; after three NO_x_-TPR (**left column**) and two isothermal (**right column**) soot oxidation cycles.

TEM images for used catalysts reveal the presence of some model soot (Printex U), after the NO_x_-TPR and isothermal soot oxidation processes (Figure 16). Thus, the deactivation of the catalysts seems to be related to the accumulation of unreacted soot that blocks the active sites. BM-C850 shows the highest amount of unreacted soot after NO_x_-TPR and isotherm tests. Note that, for BM-C600 and BM-C700, the TEM images were selected to show the degradation degree of the remaining soot, but they do not represent the amount of remaining soot. The TEM images in Figure 16 reveal that the remaining soot is in different degradation stages after the isothermal soot oxidation cycles in each catalyst. Therefore, the remaining soot in BM-C850 shows a lower degree of degradation because they hold the spherical shape expected for carbon black, while in BM-C600 and BM-C700 the remaining particles of soot have lost the spherical shape. This finding is related to the lower initial soot oxidation rate determined for BM-C850 than for BM-C600 and BM-C700 during the first isothermal cycle (see Table 3).

Summarizing, the characterization of the used catalysts proves that the deactivation is due to the accumulation of soot during successive NO_x_-TPR and isothermal soot oxidation cycles.

## 4. Conclusions

From the above-discussed results, the following conclusions can be drawn:

* The use of carbon black during the sol-gel synthesis allows the decrease in the calcination temperature required to achieve the perovskite-like structure.
* Low calcination temperature minimizes the sintering effects, allowing a lower aggregation between particles and yielding solids with lower average crystal and particle size, slightly higher BET surface area and lower diameter of macropores than the BM reference catalyst.
* BM-CX catalysts show enhanced reducibility and oxygen mobility, presenting BM-C700 catalyst as the optimum combination.
* The BM-CX series shows an improved catalytic performance regarding the BM reference catalyst for oxidation processes (NO to NO_2_ and NO_2_-assisted soot oxidation), promoting higher stability and higher CO_2_ selectivity. BM-C700 shows the best catalytic performance (i.e., the highest thermal stability and a high initial soot oxidation rate which minimizes the accumulation of soot during the soot oxidation and, consequently, the catalyst deactivation) as it features a high amount of oxygen vacancies and high reducibility that promotes the highest lattice oxygen mobility.

## Figures and Tables

**Table 1:** Structural, morphological, and textural data.

	Average Cristal Size ^a^ (nm)	Lattice Parameters ^b^		S_BET_ (m^2^/g)
		a (nm)	c (nm)	
BM-C600	26	5.693	4.809	20
BM-C700	30	5.693	4.809	21
BM-C750	30	5.697	4.806	10
BM-C800	32	5.693	4.809	8
BM-C850	32	5.693	4.809	7
BM	40	5.698	4.806	<5

^a^ by Scherrer equation using main diffraction peak BaMnO_3_ hexagonal (≈31.4°). ^b^ from main diffraction peaks BaMnO_3_ hexagonal ≈ 31.4° (110) y ≈ 25.8° (101).

**Table 2:** XPS ratios and β-O_2_ evolved during O_2_-TPD experiments.

Catalyst	O_Lattice_/(Ba + Mn)	Mn(IV)/Mn(III)	β-O_2_ (μmol/g_cat_)
Nominal	1.5 ^a^	-	-
BM-C600	1.3	0.6	60
BM-C700	1.1	0.6	510
BM-C750	1.2	0.6	445
BM-C800	1.1	0.7	190
BM-C850	1.0	0.9	210
BM	1.5	0.7	90

^a^ calculated for BaMnO_3_ composition.

**Table 3:** Initial soot oxidation rates at 450 °C and CO_2_ selectivity of BM-CX series, and references.

Catalyst	Initial Soot Oxidation Rate (10^−2^ mmol/min)		CO_2_ Selectivity (%)	
	Cycle 1	Cycle 2	Cycle 1	Cycle 2
BM-C600	3.4	3.3	92	92
BM-C700	3.7	3.2	92	92
BM-C850	3.0	3.2	86	86
BM	1.6	1.3	66	66
1 %Pt/Al_2_O_3_	2.7	2.8	100	100

**Table 4:** XPS ratios of fresh and used BM-CX catalysts in successive NO_x_-TPR and isothermal cycles.

Catalyst	O_L_/(Mn + Ba)			Mn(IV)/Mn(III)		
	NO_x_-TPR	Isothermal (450 °C)	Fresh	NO_x_-TPR	Isothermal (450 °C)	Fresh
BM-C600	0.9	0.8	1.3	0.6	0.5	0.6
BM-C700	1.1	1.2	1.1	0.6	0.6	0.6
BM-C850	1.1	0.7	1.0	0.6	0.7	0.9

**Figure A1:**
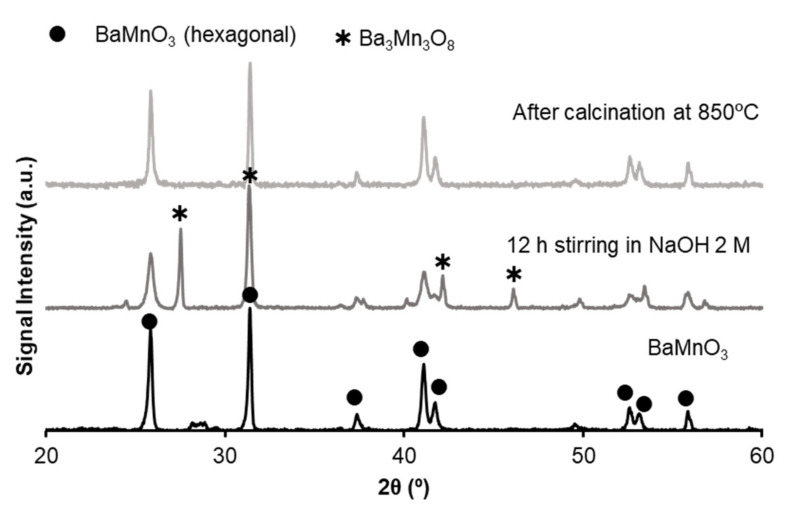
X-ray patterns for BaMnO_3_ solids: effect of alkali media and calcination step on the hexagonal perovskite structure.

**Figure A2:**
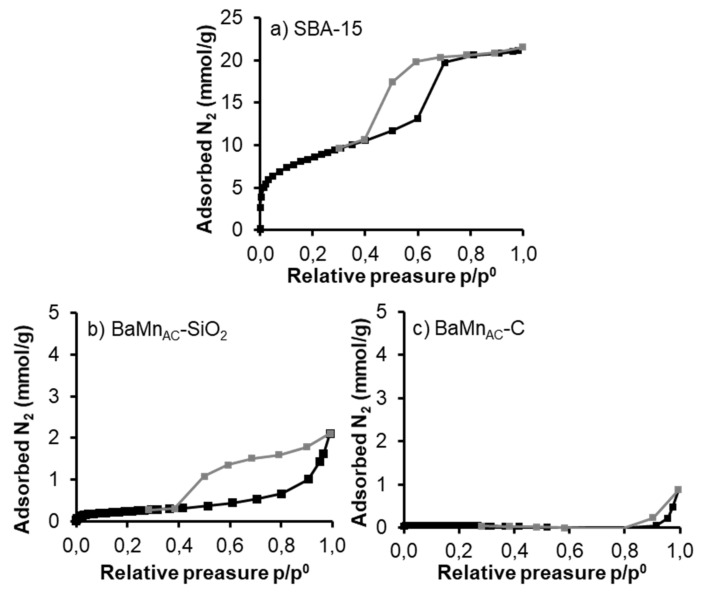
N_2_ adsorption isotherms (adsorption in black and desorption in grey) of: (**a**) the synthesized hard template (SBA-15), (**b**) the hybrid material obtained by citric acid excess route (BaMn_AC_-SiO_2_), and (**c**) the corresponding final solid after silica removal and calcination step (BaMn_AC_-C).

**Figure A3:**
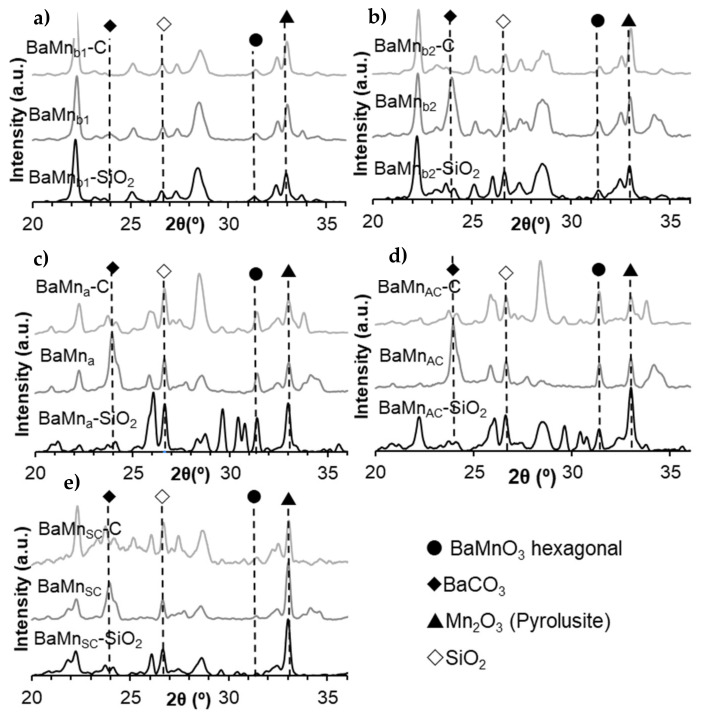
X–ray patterns of composites before (X-SiO_2_), after silica removal (X) and after calcination step (X-C) for the synthesis route indicated in Table A1: (**a**) BaMn_b1_, (**b**) BaMn_b2_, (**c**) BaMn_a_, (**d**) BaMn_AC_, and (**e**) BaMn_SC_.

**Figure A4:**
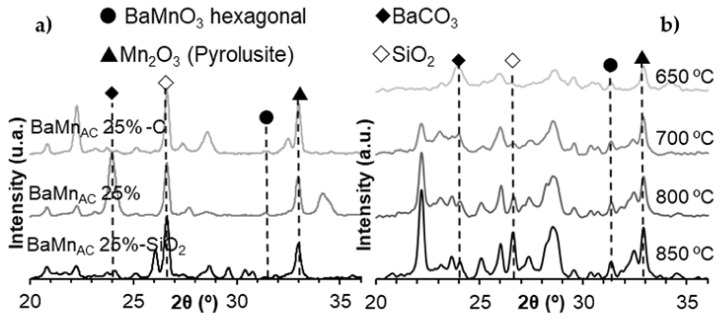
X-ray patterns of composites before (X-SiO_2_), after silica removal (X) and after calcination (X-C) for the citric acid synthesis route: (**a**) 2:1 ratio SBA-15:BaMnO_3_, and (**b**) effect of the calcination temperature on b2 synthesis.

## Data Availability

Data can be available upon request from the authors.

## Appendix A. Analyzing Different Synthesis Routes Using SBA-15 Mesoporous Silica as Hard-Template for Modified Sol-Gel Synthesis

SBA-15 mesoporous silica to be used as a hard template in the modified sol-gel synthesis method was synthesized by a soft-template route according to the literature [15,21,63]. Thus, silica is dispersed (1:10 ratio regarding deionized water) and added to the sol of the perovskite precursors (1:1 volume ratio) obtained by the route indicated in Table A1. The silica is removed from the composite by magnetic stirring in 2M NaOH [15,22,64,65] solution for 7 h at room temperature. The final solid is filtered, washed, and dried at 25 °C for 12 h. Finally, it is calcined at 850 °C (5 °C/min), because (as it is observed in X-ray patterns at Figure A1) the silica removal process damages the hexagonal perovskite structure of BaMnO_3._

In order to avoid the strong interaction between barium and silica (which causes the formation of barium silicates instead of the perovskite structure), some modifications on the conditions for obtaining the sol of precursors were carried out (Table A1). Thus, as the sol-gel synthesis process used a basic medium (in which silica presents low stability), the first approach was to decrease the pH value as follows: (i) by avoiding the addition of ammonia to the solution, so, the pH is slightly alkali (indicated by adding b2 into the nomenclature), (ii) by acidification of the sol media with HCl solution (indicated as a) and (iii) by increasing of the citric acid concentration until the total solution of the reagents (indicated as AC). Finally, a one-pot synthesis following the procedure found in the literature [66] was used (indicated as SC). The nomenclature used for samples indicates: (i) the composition of the perovskite (BaMn for BaMnO_3_); (ii) the method used in the synthesis as a subscript; (iii) for the hybrid materials perovskite, SiO_2_ is added at the end of the nomenclature and (iv) a C is added for the sample after the calcination step. The solids obtained in different steps of the synthesis were characterized by XRD and N_2_ adsorption.

**Table A1:** Synthesis conditions and nomenclature.

Synthesis *	pH ^a^	T_1_ (°C)	t_1_ (h)	T_2_ (°C)	t_2_ (h)	Calcination	Nomenclature
Basic-1 (b-1)	NH_3_	- ^b^	4	80	- ^c^	3 °C/min-500 °C-5 h 5 °C/min-850 °C-6 h	BaMn_b-1_-SiO_2_
Basic-2 (b-2)	NH_3_	65	5	90	48		BaMn_b-2_-SiO_2_
Acid (a)	HCl	- ^b^	4	80	- ^c^		BaMn_a_-SiO_2_
Citric Acid Excess (AC)	- ^d^	- ^b^	4	80	- ^c^		BaMn_a_-SiO_2_
One-Pot (SC)	-	65	5	80	24		BaMn_SC_-SiO_2_

* Calcination conditions: ^a^ used reagent to adjust the pH of the sol, ^b^ magnetic stirring at room temperature, ^c^ the indicated temperature is kept until dryness, ^d^ if the reagents of the precursors do not dissolve properly the amount of citric acid is increased.

Figure A2 shows that, as it is expected [19,21], the synthesized SBA-15 silica shows a type IV isotherm with a hysteresis loop which is characteristic of porous solids with a wide proportion of mesoporous [67]. As an example, Figure A2 presents the N_2_ adsorption isotherms of the samples obtained by the citric acid excess route (AC) at different steps: before (BM_AC_-SiO_2_) and after (BM_AC_) the silica removal step, and after the calcination step (BM_AC_-C). All the samples show a type V isotherm, which corresponds to non-porous solids. BET surface areas, shown in Table A2, are lower than expected for hybrid materials [14,21,22,66] and also for solids after the removal of the silica template [15,19,64,66]. Therefore, the porous structure of silica could be blocked or, the structure of SBA-15 was modified during the synthesis process.

**Table A2:** Specific surface area (S_BET_) of composite materials before and after silica removal and calcination.

Hard-Template		S_BET_ (m^2^/g)	
SBA-15		699	
Composite	S_BET_ (m^2^/g)	Composite	S_BET_ (m^2^/g)
BaMnO_3_ (alkali media)	≈5	BaMnO_3_ (acid media)	≈3
BaMn_b-1_-SiO_2_	14	BaMn_a_-SiO_2_	11
BaMn_b-2_-SiO_2_	28	BaMn_AC_-SiO_2_	20
BaMn_b-1-_-C	10	BaMn_a_-C	9
BaMn_b-2_-C	≈4	BaMn_AC_-C	≈4

Figure A3 presents the X-ray patterns for the samples in different steps of the synthesis routes indicated in Table A1. The X-ray patterns show the range from 20° to 36° 2θ values because the main peak of BaMnO_3_ hexagonal perovskite is located at c.a. 31.4°. Note that all the non-assigned diffraction peaks (which hinders the interpretation of the X-ray patterns) correspond to several barium silicates and that the samples obtained by the synthesis in alkali media (b1 and b2) show a higher amount of barium silicates because silica is less stable at high pH values. Thus, it is concluded that any of the synthesis procedures used allow the formation of the perovskite structure as the main crystal phase.

In order to confirm that the suggested synthesis methods do not allow obtaining the perovskite structure as main crystal phase, two additional modifications of the synthesis method were carried out: (i) reducing the proportion SBA-15:BaMnO_3_ (from 1:1 to 2:1) for citric acid excess synthesis route, because it yields the higher proportion of BaMnO_3_ hexagonal (although it is a minority phase) and (ii) testing different calcination temperatures for b2 synthesis. The samples were characterized by the XRD technique, and the corresponding X-ray patterns are gathered in Figure A4, which shows only the range of 2θ° values from 20° to 36°, and in which all the non-identified peaks correspond to different types of barium silicates. The results reveal that the use of a lower amount of BaMnO_3_ precursors hinders the synthesis of the perovskite structure and that the formation of the BaMnO_3_ hexagonal perovskite begins at 600 °C.

Summing up, to synthesize a mesoporous BaMnO_3_ hexagonal perovskite, SBA-15 is not valid as a hard template. However, the use of a hard template seems to reduce the calcination temperature required to form the perovskite structure.

## Appendix B. Data Related to the Removal of Carbon Black Remained after Synthesis

Data in Table A3 indicate that the total weight loss corresponds to the evolved CO_2_ (inside Figure A5), which mainly comes from the removal of the remaining carbon black used as hard template and that the percentage of remaining carbon black decreases as the calcination increases.

**Table A3:** Total weight loss calculated by TG and MS data.

	Total Weight Loss (TG) (%)	Weight Loss Due to CO_2_ Emission (m/z 44 MS) (%)
BM-C600	4	3
BM-C700	4	3
BM-C750	3	2
BM-C800	2	1
BM-C850	2	1
BM	1	1
